# Supplementary material for: Efficacy of a Six-Week Dispersed Wingate-Cycle Training Protocol on Peak Aerobic Power, Leg Strength, Insulin Sensitivity, Blood Lipids and Quality of Life in Healthy Adults
Source: Int J Environ Res Public Health. 2020 Jul 6;17(13):4860. doi: 10.3390/ijerph17134860 (PMC7369806; doi:10.3390/ijerph17134860)
Supplement: Supplementary file 1 [file ijerph-17-04860-s001.pdf]

Supplementary Table

Mean pre and post-intervention results in the Control (n = 17) and Intervention (n = 16) groups

		Pre- Intervention	Post-Intervention
Body mass (kg)	CON	69.0 ± 14.7	69.3 ± 15.2
	INT	69.3 ± 14.2	69.2 ± 13.7
Body fat (%)	CON	26.3 ± 9.8	25.5 ± 9.8
	INT	24.9 ± 7.2	24.8 ± 7.8
Resting Systolic BP (mmHg)	CON	120 ± 14	119 ± 14
	INT	117 ± 13	113 ± 13
Resting Diastolic BP (mmHg)	CON	79 ± 11	77 ± 11
	INT	76 ± 8	74 ± 9
Resting HR ($b \cdot min^{-1}$)	CON	65 ± 10	65 ± 10
	INT	63 ± 10	66 ± 9
VO_{2peak} ($L \cdot min^{-1}$)	CON	2.50 ± 0.68	2.53 ± 0.75
	INT	2.58 ± 0.86	2.75 ± 0.78
VO_{2peak} ($ml \cdot kg^{-1} \cdot min^{-1}$)	CON	36.9 ± 9.6	37.1 ± 10.1
	INT	36.7 ± 6.7	39.4 ± 5.7
MVC extension $30° \cdot s^{-1}$ (Nm)	CON	158 ± 42	145 ± 40
	INT	168 ± 59	172 ± 47
MVC flexion $30° \cdot s^{-1}$ (Nm)	CON	91 ± 31	93 ± 28
	INT	86 ± 25	96 ± 25
MVC extension $300° \cdot s^{-1}$ (Nm)	CON	80 ± 27	79 ± 23
	INT	87 ± 31	84 ± 24
MVC flexion $300° \cdot s^{-1}$ (Nm)	CON	55 ± 23	58 ± 21
	INT	48 ± 16	59 ± 18
Total Cholesterol ($mmol \cdot l^{-1}$)	CON	4.97 ± 0.75	4.88 ± 0.52
	INT	4.74 ± 0.93	4.60 ± 0.81
Triglycerides ($mmol \cdot l^{-1}$)	CON	2.00 ± 1.06	2.06 ± 1.36
	INT	1.79 ± 0.99	1.78 ± 1.07

HDL (mmol·l^{-1})	CON	1.68 ± 0.46	1.64 ± 039
	INT	1.60 ± 0.37	1.60 ± 0.35
LDL (mmol·l^{-1})	CON	2.87 ± 0.71	2.84 ± 0.61
	INT	2.77 ± 0.75	2.65 ± 0.66
Fasting Glucose (mmol·l^{-1})	CON	4.79 ± 0.57	4.71 ± 0.46
	INT	4.56 ± 0.42	4.51 ± 0.36
Fasting Insulin (mmol·l^{-1})	CON	5.96 ± 3.70	6.61 ± 4.80
	INT	4.68 ± 3.16	6.43 ± 4.02
Insulin resistance – HOMA (au)	CON	1.32 ± 0.89	1.45 ± 1.16
	INT	0.96 ± 0.71	1.32 ± 0.88
Insulin sensitivity – QUICKI (au)	CON	0.39 ± 0.07	0.38 ± 0.05
	INT	0.39 ± 0.06	0.38 ± 0.04

CON = control or non-training group; INT = Intervention or training group; BP = blood pressure; HR = heart rate; VO_{2peak} = peak oxygen uptake; MVC = maximal voluntary contraction; HDL = High-density lipoprotein; LDL = low-density lipoprotein; HOMA = Homeostatic model assessment; QUICKI = Quantitative insulin-sensitivity check index; au = arbitrary unit.
